# Nine-Axis Sensor for Athlete Physical Training Load Characteristics

**DOI:** 10.1155/2022/1538331

**Published:** 2022-01-15

**Authors:** Meifu Liang, Ningning Zhao, Yamei Li

**Affiliations:** ^1^China Institute of Sport Science, Beijing 100010, China; ^2^Hebei Institute of Sports Science, Shijiazhuang, Hebei 050000, China; ^3^Hebei University of Chinese Medicine, Shijiazhuang, Hebei 050000, China

## Abstract

In order to understand the characteristic data of athletes' training load, a method based on nine-axis sensor was proposed. Twenty-seven male college athletes were tested twice with a time interval of more than 48 hours. In part 1, participants take the 1 Repetition Maximum (1RM) test. The results show that maximum strength is one of the basic factors to develop the output power of athletes. In the process of skeletal muscle contraction, the curve of speed, force, and power is closely related. When the external load is 10%∼70%, the average power increases with the increase in the average force, it increases with the decrease in the average speed, and at 70%1RM, the average power reaches the peak and then decreases at an inflection point. It is proved that the accurate weight ratio of strength training is the basis of winning athletes, the focus of high level physical coach, and the premise of scientific sports training.

## 1. Introduction

With the continuous reduction of the volume of electronic hardware and the improvement of chip computing speed, more and more domestic and foreign researchers have begun to study the motion posture of the human body [1]. The nine-axis inertial sensor is composed of three-axis acceleration, three-axis gyroscope, and three-axis magnetometer and is first applied in the military space field [2]. With the development of micromechanical and electronic technology, the volume of inertial sensors is getting smaller and smaller, the cost is getting lower and lower, and the chip processing speed is getting faster and faster, and they begin to be applied in the field of motion attitude recognition [3]. Domestic-related research is still focused on quantitative evaluation algorithm research, and commercial products have not been reported. Many foreign companies have carried out research and development of PD quantitative evaluation products, such as the Research Kit launched by Apple, which aims to provide corresponding interfaces for PD patient data collection [4]. Kinesia 360, a quantitative evaluation system for human motor function, was developed by Cleveland Medical Instrument Company in the United States, which adopted accelerometer and gyroscope sensors to record motion information; the device was worn on the wrist and fingers of patients to dynamically monitor upper limb motor dysfunction [5]. Global Kinetics, an Australian technology company, also has a related product, which can collect data on the bradykinesia symptoms of PD patients for 10 days, so that doctors can fine-tune therapeutic drugs [6]. The vibration signal measurement methods used in the above products are total accelerometer method, optical measurement, and inertial measurement. The displacement signal can be obtained by multiple integration of the acceleration signal measured by the total accelerometer, but the information obtained is limited. Different from the total accelerometer method, optical measurement and inertial measurement can obtain the motion information of the tremor in three directions in space [7]. Comparatively speaking, the optical measurement system is inconvenient in practical application, while the inertial measurement system has the advantages of small size, low cost, and easy to use and has a broader development prospect. The existing inertial measurement usually uses three-axis accelerometer and three-axis gyroscope to realize attitude demodulation, but there is an integral accumulated error in the long-term operation, which is manifested as the baseline drift of the measured angle [8]. To this end, a triaxial magnetometer was added to make the correction. After the nine-axis parameters are fused, the long-time attitude demodulation accuracy can be greatly improved. In recent years, with the development of MEMS technology, nine-axis sensors have achieved integration, miniaturization, and micropower consumption and are entering the market of motion sensing applications such as indoor navigation of mobile devices, gesture recognition, and laptop screen flipping angle measurement in a large scale; applying them to tremor detection can maximize its effect and achieve better results [9].

In recent years, the domestic-related research is still focused on the quantitative evaluation algorithm research, and commercial products have not been reported. Many foreign companies have developed PD quantitative evaluation products, such as the Research Kit launched by Apple Inc., which aims to provide corresponding interfaces for PD patient data collection. Kinesia 360, a quantitative evaluation system for human motor function, was developed by Cleveland Medical Instrument Company in the United States, which adopted accelerometer and gyroscope sensors to record motion information; the device was worn on the wrist and fingers of patients to dynamically monitor upper limb motor dysfunction [10]. Global Kinetics, an Australian technology company, also has a product that collects 10 days' worth of data on the symptoms of bradykinesia in PD patients, allowing doctors to fine-tune treatments. Chen et al. designed a nine-axis sensor-based abnormal behavior detection system. The system uses a triaxial acceleration sensor and a triaxial gyroscope sensor to collect the acceleration and angular velocity of human posture. A triaxial magnetometer is used to assist in positioning. Sensors are used to collect heart rate, temperature, and human characteristic parameters. SIM900A module GSM/GPRS is used for geographic information collection and SMS alarm, and the corresponding geographic location and collection and conversion parameters are sent to the guardian, so as to realize the detection of abnormal behaviors [11]. Liet al. studied a swimming posture recognition method based on the nine-axis sensor, the original data were collected by the nine-axis sensor, the quaternion is calculated by the equivalent rotation vector method, and the dimension is reduced by the spherical pole projection and vertical projection of the quaternion; the feature information is extracted on the two-dimensional plane, and the swimming posture is recognized by the discriminant model based on classifier. The invention can accurately identify the swimming posture [12]. Shrivastava et al. studied a crane control system based on nine-axis sensors; the crane has a main beam 1, a trolley 2 mounted on the main beam 1, and a hook 3 suspended below the trolley; the left end of the main beam is arranged at the left end of the main beam 4, the left walking wheel 5, and the left driving mechanism 6 supporting the left end of the left beam 4; the right end of the main beam is arranged at the right end of the main beam 1 and 7, supporting the right end beam 7, the right traveling wheel 8, and the right drive mechanism 9; the left end of the main beam 1 is provided with a 19-axis sensor, the right end of the main beam 1 is provided with a second and ninth-axis sensor; the left end beam 4 and the right end beam 7 have the same structure; and the invention solves the asynchronism problem caused by mechanical wear and other reasons of the multimotor system running for a long time [13]. Based on the current research, a method based on the nine-axis sensor is proposed. Twenty-seven male college athletes were tested twice with a time interval of more than 48 hours. In part 1, participants take the 1 Repetition Maximum (1RM) test. In part 2, subjects are tested for maximum power output of bench press throw and half squat under different loads. OPL of different strength training means is determined, OPL output power characteristics are explored, and OPL-related influencing factors are analyzed in order to promote OPL strength training practice, to achieve scientific sports training and scientific preparation for athletes to provide some scientific support. The Mini IMU (version: 4.3.14) software is used for data collection of all test indicators.

## 2. Research Objects and Methods

### 2.1. Research Object

There are 27 male college athletes in sprint, all of whom have the experience of half squat and bench press. All subjects were free of various visceral diseases, normal liver and kidney function, and no bad habits. The subjects were required to be caffeine-free for 3 hours prior to the test, to have not performed intense resistance exercise for 24 hours, and to have no contrainditions such as lower limb joint injury (open and closed), cardiovascular disease, skin allergy, and hernia in the past 3 months. Basic information of the subjects is shown in Table 1. All subjects were informed of the potential risks of participating in the experiment in advance and signed the informed consent [14].

### 2.2. Experimental Method

#### 2.2.1. Experimental Equipment

Experimental equipment includes the following: 1 nine-axis Bluetooth attitude sensor WIT-Motion (BWT901CL); 1 laptop; 2 sets of Smith rack (including standard barbell bar, barbell plate, and fixed spring clip); 1 stopwatch 1; 1 roll of scotch tape; 1 external camera; and 1 spare power supply [15].

#### 2.2.2. Experimental Process

This experiment was divided into two parts, and two tests were conducted with a time interval of more than 48 hours. In part 1, participants take the 1 Repetition Maximum (1RM) test. In part 2, subjects were tested for maximum output power of bench press throw and half squat under different loads [16].

Before the experiment, the height and weight of the subjects were measured and recorded, the experiment equipment and test process were shown to the subjects, the test content and action requirements were explained to the subjects, and the test movement and test process were simulated. Half squat and flat bench press were tested by 1RM using NSCA. The maximum output power test of half squat jump and bench press throw was carried out under 10%, 30%, 50%, 70%, and 90%1RM loads, effective data should be collected once for each load level, and the interval between groups was 3 to 5 minutes [17–19].

Before each test, subjects performed 15 min of routine warm-up activities, including 10 min of moderate intensity jogging, 5 min of dynamic lower limb stretching, 3–5 times of nonweight-bearing squat jumping and bench press throwing exercises, and 3 min of rest before the test. The 1RM test and the maximum output power test were carried out on the Smith rack, with protection personnel on both sides [20].

#### 2.2.3. Data Collection Process

The software Mini IMU (version: 4.3.14) was used to collect data, the baud rate was set to 115200 bit/s, the return rate was set to 100 Hz, the acceleration range was set to 0–8 G gravity acceleration, the zero offset value of the acceleration was calculated, and the parameters were written, the output content was time and acceleration. The specific collection process is as follows:

- Basic information of registered athletes is collected.
- The experimental operator shall make relevant settings in the acquisition software to check that the device signals are normal.
- After the test load is set, the “prepare” password is issued to the subjects; after the subjects get on the Smith rack, the operator will return the control software to the zero setting.
- When the subjects are ready, the experiment operator will trigger the collection in the computer collection window and issue the command “3, 2, 1, start”; after the sampling, the subjects will leave the Smith rack and save the data.

#### 2.2.4. Data Processing

The output power, speed, and force values are calculated according to the time, acceleration, and training load. The specific calculation formula is as follows: *V*_*i*_=*V*_*i*−1_+*a*_*i*_ × *t*_*i*_, *F*_*i*_=*m*_*i*_ × (*a*_*i*_+*g*), and *P*_*i*_=*F*_*i*_ × *V*_*i*_ where *A* is the vertical acceleration, *V* is the instantaneous velocity, *P* is the output power, *m* is the training load, *g* is the gravity acceleration (10 m/s^2^), and the initial velocity *V*_0_ = 0 m/s.

## 3. Research Results

### 3.1. Determination of OPL of Squat Jump

**Figure 1:**
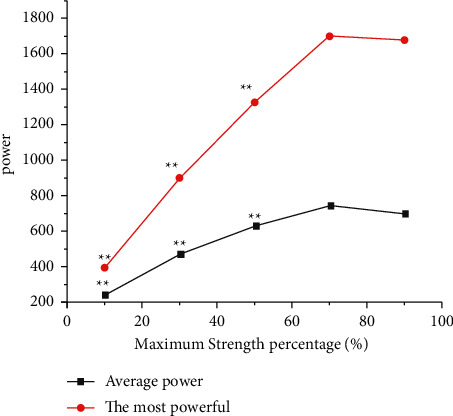
Output power characteristics of squats under different loads.

The output power characteristics of horizontal push and tossing under different loads are as shown in Figure 1. When the load intensity was 70%1RM, the average output power and the maximum output power were significantly higher than that of 10%1RM, 30%1RM, and 50%1RM (*P* < 0.01) but had no significant difference with that of 90%1RM (*P* > 0.05).

### 3.2. Correlation Analysis of OPL Variables in Squat Jump

As can be seen from Table 2, there is a very high correlation between average power and maximum power (*r* = 0.94; *P* < 0.01) and a high correlation between output power and maximum speed (*r* = 0.84∼0.87; *P* < 0.01).

### 3.3. Determination of OPL for Bench Press Throwing

**Figure 2:**
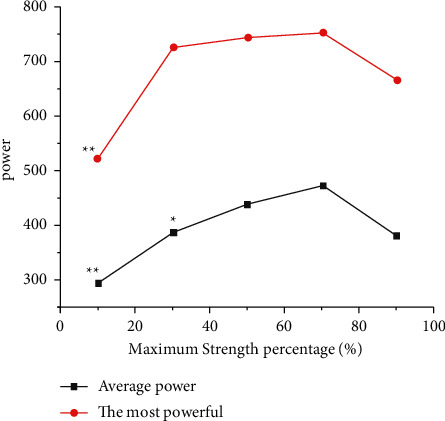
Output power characteristics of bench push and throw under different loads.

The output power characteristics of horizontal push and tossing under different loads are as shown in Figure 2. The average output power and the maximum output power of bench press throwing increased with the increase in training load intensity, and the output power reached the peak when the training load intensity was 70%1RM; that is, the OPL of bench press throwing was 70%1RM. When the load intensity was 70%1RM, the average output power was significantly higher than that of 10%1RM (*P* < 0.01), 30%1RM (*P* < 0.05), and 90%1RM (*P* < 0.01), but there was no significant difference between the average output power and that of 50%1RM (*P* > 0.05). When the load intensity was 70%1RM, the maximum output power was significantly higher than 10%1RM (*P* < 0.01) but had no significant difference with 30%1RM, 50%1RM, and 90%1RM (*P* > 0.05).

### 3.4. Correlation Analysis of OPL Variables in Bench Press Throwing

It can be seen from Table 3 that average power is highly correlated with maximum power, average speed, maximum speed, and maximum force (*r* = 0.7–0.84; *P* < 0.01), moderately correlated with average force and 1RM (*r* = 0.77–0.7; *P* < 0.01), and mildly correlated with body weight and training years (*r* = 0.4–0.46; *P* < 0.05). The maximum power was highly correlated with maximum speed (*r* = 0.74; *P* < 0.01), moderately correlated with average speed, average force, maximum power, and bench press 1RM (*r* = 0.57–0.67; *P* < 0.01), and slightly correlated with training years (*r* = 0.44; *P* < 0.05).

**Figure 3:**
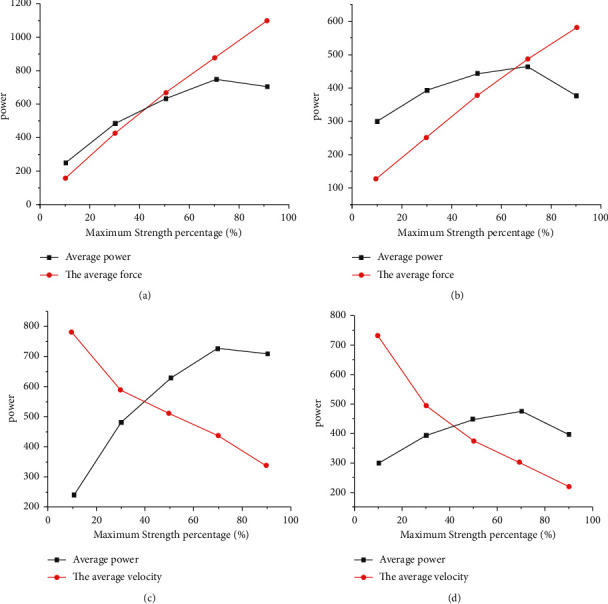
Power-speed and power-force curves of half squat jump and bench press throw.

Maximum strength is one of the basic elements to develop the output power of athletes, as shown in Figure 3; the curve of speed, force, and power is closely related in the process of skeletal muscle contraction; when the external load is between 10% and 70%, the average power increases with the increase in the average force and the decrease in the average speed; at 70%1RM, the average power reaches the peak and then decreases at an inflection point.

## 4. Discussion

### 4.1. Wireless Communication Program Design

Because this system is to realize one-to-many wireless data communication, that is, a central main control device (PC) communicates with multiple nine-axis sensor sensor detection data acquisition equipment and all wireless devices work on the same wireless channel, and they can send and receive data on this wireless channel, how to allocate wireless channel resources reasonably and efficiently is a very important problem. In the polling mode, the central main control computer sends the inquiry signal to each physical testing equipment in sequence 1; the data acquisition device of the corresponding nine-axis inertial sensor sends real-time data acquisition and processing after receiving the inquiry sign all; after the transmission is completed, a one-bit end signal is sent; and the central control computer stores and processes the data after receiving it. In this way, the one-to-one communication between the main control device and the sensor device is completed. Then, the central control device sends the inquiry signal to the next nine-axis inertial sensor data acquisition device, and so on, to complete the data communication to all nine-axis inertial sensor data acquisition devices, that is, the realization of one-to-many communication.

### 4.2. Computer Software Design

The main function of the computer software is to collect the motion information data of the nine-axis inertial sensor through Socket communication. In data acquisition, the computer software is the main server and the sensor is the client. With the client connected to the router, the port number of the server is set to 8000, and the server obtains the preset LOCAL IP address 192.168.1.51; after entering the tester number, click “start,” the server sends the test protocol to the client, and the client sends the data after receiving the protocol. In the process of Socket communication, TCP, a connection-oriented protocol, is used when data transmission is involved. Its main purpose is to provide a large number of data transmission and ensure that the transmission is correct. A three-way handshake is used to establish a TCP connection. That is, three packets are exchanged.

## 5. Conclusions

In order to understand the characteristic data of athletes' training load, a method based on nine-axis sensor was proposed. Twenty-seven male college athletes were tested twice with a time interval of more than 48 hours. In part 1, participants take the 1 Repetition Maximum (1RM) test. in part 2, subjects are tested for maximum power output of bench press throw and half squat under different loads. The OPL of different strength training methods was determined, the output power characteristics of OPL were explored, and the relevant influencing factors of OPL were analyzed in order to promote the practicalization of OPL strength training, realize the scientific sports training, and provide some scientific support for the scientific preparation of athletes. The data obtained from all test indicators are Mini IMU (Version number: 4.3.14) data collection by software. The results show that maximum strength is one of the basic factors to develop the power output of athletes (Baker, 2001). As shown in Figure 3, the curves of speed, force, and power are closely related in the process of skeletal muscle contraction; when the external load is between 10% and 70%, the average power increases with the increase in the average force; it increases with the decrease in the average speed; and at 70%1RM, the average power reaches the peak and then decreases at an inflection point. It is proved that the accurate weight ratio of strength training is the basis of winning athletes, the focus of high level physical coach, and the premise of scientific sports training.

## Figures and Tables

**Table 1:** Basic information of subjects (*N* = 27).

Age	Height (cm)	Weight (kg)	Years of training (year)	Crouch weight 1RM	Bench press weight 1RM
21.48 ± 2.24	179.41 ± 5.29	69.88 ± 5.80	3.65 ± 1.90	115.74 ± 18.54	65.56 ± 10.13

*Note*. 1RM is the maximum strength.

**Table 2:** Mean, standard deviation, and correlation coefficient of each variable.

Variable	M	SD	1	2	3	4	5	6	7	8	9
(1) Average power	740.81	217.33	1								
(2) The most powerful	1689.31	547.6	0.94^*∗∗*^	1							
(3) The average velocity	0.89	0.27	0.68^*∗∗*^	0.55^*∗∗*^	1						
(4) Maximum speed	1.65	0.41	0.87^*∗∗*^	0.84^*∗∗*^	0.78^*∗∗*^	1					
(5) The average force	881.03	135.73	0.50^*∗∗*^	0.61^*∗∗*^	−0.09	0.11	1				
(6) The most strongly	1091.2	166.18	0.43^*∗∗*^	0.59^*∗∗*^	−0.14	−0.10	0.91^*∗∗*^	1			
(7) Weight	69.88	5.80	0.26	0.35	0.03	0.18	0.44^*∗∗*^	0.32	1		
(8) Years of training	3.61	1.95	0.35	0.41^*∗*^	0.20	0.21	0.38	0.46^*∗*^	0.10	1	
(9) 1RM	115.74	18.54	0.40^*∗*^	0.48^*∗*^	−0.18	−0.01	0.93^*∗∗*^	0.83^*∗∗*^	0.36	0.25	1

Note: ^*∗*^means significance at the level of 0.05; ^*∗∗*^ means significance below the level of 0.01.

**Table 3:** Mean, standard deviation, and correlation coefficient of each variable in bench press.

Variable	M	SD	1	2	3	4	5	6	7	8	9
(1) Average power	473.56	115.80	1								
(2) The most powerful	753.48	211.22	0.76^*∗∗*^	1							
(3) The average velocity	0.99	0.18	0.80^*∗∗*^	0.59^*∗∗*^	1						
(4) Maximum speed	1.46	0.32	0.84^*∗∗*^	0.74^*∗∗*^	0.90^*∗∗*^	1					
(5) The average force	490.96	72.00	0.65^*∗∗*^	0.54^*∗∗*^	0.07	0.25	1				
(6) The most strongly	614.61	100.44	0.70^*∗∗*^	0.67^*∗∗*^	0.23	0.38^*∗*^	0.87^*∗∗*^	1			
(7) Weight	69.88	5.80	0.46^*∗*^	0.22	0.10	0.09	0.65^*∗∗*^	0.56^*∗∗*^	1		
(8) Years of training	3.62	1.95	0.40^*∗*^	0.44^*∗*^	0.14	0.31	0.49^*∗∗*^	0.61^*∗∗*^	0.09	1	
(9) 1RM	65.56	10.13	0.57^*∗∗*^	0.57^*∗∗*^	0	0.19	0.96^*∗∗*^	0.89^*∗∗*^	0.61^*∗∗*^	0.48^*∗*^	1

## Data Availability

The data used to support the findings of this study are available from the corresponding author upon request.
